# Entrepreneurship: Tenacity, Future Self-Continuity, and Inter-Temporal Risky Choice

**DOI:** 10.3389/fpsyg.2020.01647

**Published:** 2020-08-07

**Authors:** Xueyun Zeng, Yuting Ouyang

**Affiliations:** ^1^School of Economics and Management, Beijing University of Posts and Telecommunications, Beijing, China; ^2^School of Sociology, China University of Political Science and Law, Beijing, China

**Keywords:** entrepreneurship, tenacity, future self-continuity, risky choice, inter-temporal choice

## Abstract

This study examines entrepreneurship. It focuses on the effect of tenacity and future self-continuity (FSC) on inter-temporal risky choice in the entrepreneurial context. A total of 129 Chinese undergraduates participated in this survey. The results formulate that tenacity positively correlates with the risky choices and inter-temporal risky choices, in which commitment, endurance, and challenge play a major role. Meanwhile, FSC predicts the risk-return of the subjects. Higher FSC corresponds to higher expected inter-temporal risk-return. Furthermore, the multivariable regression analysis shows that there is a reciprocal effect when tenacity and FSC work together on subjects’ inter-temporal risky decision-making. FSC slightly mitigates both the pursuit of risky-return and the tolerance of time delay for the subjects with high tenacity. This implies that their worthwhile goal is to seek smooth income rather than to pursue an extreme high risk-return. These findings extend the research on personality, choice, and entrepreneurship and provide a guiding significance to the start-up.

## Introduction

In the past few decades, entrepreneurship has become one of the main options for students when they graduate (Peterman and Kennedy, 2003; Pihie, 2009; Ekpoh and Edet, 2011). Entrepreneurial enthusiasm comes from a variety of drivers. Numerous universities provide platforms and funding for graduate entrepreneurship (Sihombing, 2012; Hou et al., 2019; Wang et al., 2019). Many countries fund college start-up projects, in order to promote economic development. Entrepreneurship causes a wide range of social concerns for government, organizations or individuals. Correspondingly, entrepreneurship is receiving attention in different disciplines, such as economics, sociology, and psychology (Tian et al., 2018; Scotter and Garg, 2019). Since Shaver and Scott (1992) pointed out that choice affects behavior and the entrepreneur’s own factors affect choice, a number of studies have been conducted on the topic of choice and personality related to entrepreneurial behavior. Among them, the role of tenacity in entrepreneurship has aroused researchers’ interest. The effect of tenacity on entrepreneurial choices is a key concern of this study. The influence of future self-continuity (FSC) and its co-effect with tenacity on inter-temporal risky choice are also the focus of this study. In the entrepreneurial context, which is preferred: the start-up with a long delay and a high risk-return, or the start-up with a relatively low risky-return but short delay? These relationships are important and unknown and need to be explained. They will be main aspects of this study.

### Theoretical Background

#### Tenacity and Entrepreneurship

Researchers have done a lot of work on the establishment of enterprises. Some studies find that personality characteristics influence entrepreneurial intentions and ability (Krueger and Brazeal, 1994; Rauch and Frese, 2007; Desti and Kumar, 2008; Miller, 2014; Wu et al., 2019), such as tenacity, positive mood, ambition, goal-striving, high energy, high honesty/integrity, self-confidence, and creativity. Based on this prior research, a great deal needs to be done to explore the role of specific traits on choices and decision-making at a given stage of entrepreneurship.

Tenacity derives from the Latin phrase “ten c-, ten x,” which originally meant “holding fast, tenacious.” Later, it evolved into middle English “Tenacite.” Now, tenacity denotes never giving up easily and being determined. This urges people to stick to their ideals. Tenacity is always interpreted to be an admirable trait in applied psychology. “Tenacity, or perseverance, is a trait that involves sustaining goal-directed action and energy even when faced with obstacles” (Baum and Locke, 2004).

It is generally known that tenacity has become recognized as important for success. On the one hand, some of the earlier articles mentioned that tenacity is associated with successful leadership (Bass and Stogdill, 1990; House and Shamir, 1993; Locke, 2000). An entrepreneur is a leader in enterprise who leads a team to accomplish difficult tasks, such as marketing and new product development. On the other hand, some researchers have seen tenacity as an entrepreneurial trait for start-up. This trait is common in different entrepreneurial situations, such as the success of marketing (Tadajewski and Jones, 2017), the angel investors (Murnieks et al., 2016), and the manufacturing venture (Scotter and Garg, 2019). Baum and Locke (2004) demonstrated the mediating effect of tenacity between the CEOs’ new resource skill and venture growths in a longitudinal study of a single industry. Murnieks et al. (2016) found that angel investors valued the tenacity and passion of entrepreneurs. In addition, the entrepreneurial experience of angels positively moderated the value provided by passion and tenacity. Scotter and Garg (2019) found that both entrepreneurial self-efficacy and tenacity impact subsequent entrepreneurial persistence in new venture creation. Moreover, tenacity seems to matter more for continuing to pursue new ventures than self-efficacy in manufacturing industry contexts (Scotter and Garg, 2019). In a word, tenacity is crucial in starting a business (Tadajewski and Jones, 2017).

It is worth mentioning that there is much room for improvement in the understanding of tenacity because of the limited literature and the lack of measurement tools. While tenacity is generally concerned and recognized, as Baum and Locke (2004) argued, it is rarely studied quantitatively. Recently, this does not seem to have changed much. Tenacity is often mentioned in entrepreneurs’ narratives, but it is rarely seriously studied to explore the psychological mechanism of tenacity in corporate development or entrepreneurship. Moreover, how to assess the diversity of people’s tenacity remains almost a blank on entrepreneurship.

#### Inter-Temporal Choice and Risky Choice

Choice or decision-making is another psychological traditional discipline (Shaver and Scott, 1992). The forces and principles behind the decision-making behaviors of humans have been widely studied by researchers (Thaler, 2008; Ariely, 2009). The impact of many internal or external factors on economic decision-making is considered, such as cognitive processes (Mata et al., 2008), emotion (Sun et al., 2015), and poverty (Carvalho et al., 2016). The parameters used to measure the “trait” of common economic decisions include time discounting, loss aversion, expected return, and probability. In this respect, two basic economic decisions – inter-temporal choice and risky choice – are proposed, which are related to time discounting and loss aversion, respectively.

Inter-temporal choice is a trade-off of today’s small gains compared with large delayed gains. The time discount rates used to test the value of large delayed gains are generally related to Discount Utility Theory (Samuelson, 1937). Researchers examined the preference of inter-temporal choice by calculating the time-delay discount rate based on Discount Utility Theory. In order to obtain basic results, the return is assumed to be certain. So, the usual scenario is savings or potential rewards. In this context, the future moment is not too far away. As a result, there is a great deal of literature using the Quasi-Hyperbolic Discounting Model to calculate the time discount rate (e.g., Laibson, 1997; Lades, 2012; Plambeck and Wang, 2013).

Risky choice is also a dilemma, involving a trade-off between a high probability but small risky-return and a low probability but large risky-return. For instance, are you choosing between a relatively stable saving or a more risky but higher-return investment? Are you choosing employment for a certain income, or starting your business for an uncertain profit? Is it the choice to follow a business model for a relatively certain market, or to create a new business model for an untapped market? To quantify the process of risk decision-making, both Expectation Model (e.g., Prelec and Loewenstein, 1991; Epper et al., 2011) and Heuristic (Brandstätter et al., 2006) are used. In Excepted Utility Theory (EUT), the traditional expectation model refers to the normative and descriptive theories of risky choice that support the view that people make risky choices based on individual expectations. But, it has been found that there are two similar anomalies that violate the conventional theoretical models in both inter-temporal choices and risky choices (Prelec and Loewenstein, 1991; Loewenstein and Prelec, 1992; Sun and Li, 2010).

There are several studies that combined inter-temporal choice with risky choice, considering the riskiness of inter-temporal decision-making in economic behavior. Some studies found that the probability discount also follow the hyperbolic discounting model (Green and Myerson, 2004), with a significant positive correlation between the probability discount and the time discount of the same subject (Myerson et al., 2003; Jones and Rachlin, 2009). Keren and Roelofsma (1995) reported that inter-temporal choice that introduced uncertainty reduced the discount rate of future rewards, just as it would increase time delays. It can be seen that inter-temporal choice and risky choice have some relatively similarity (Ahlbrecht and Weber, 1997; Weber and Chapman, 2005). However, other studies suggest that risky choice and inter-temporal choice follow different psychological processes. For instance, the subjects are less patient in inter-temporal choices with risk (Anderson and Stafford, 2008), and the individuals are more impatient for gambles than for certain outcomes in inter-temporal choices (Öncüler, 2000). However, Sun and Li (2010) argued that their findings were inconsistent with the single-process view of time delay and risk. It can be seen that there is different interpretation on the influence mechanism of inter-temporal choice and risky choice.

Considering the difference and similarity of risky choice and inter-temporal choice in the psychological process, what is lacking is the analysis of combined effects in specific situations. In fact, most choices involve both risks and delays, and the certainty assumptions about future events are sometimes unrealistic. Researchers need to put forward the decision model of both inter-temporal and risky choice and carry out empirical research to further explore the psychological process of decision-making (Konstantinidis et al., 2020). Specific to this study, entrepreneurship or not is essentially a risky choice across time. Therefore, it is a risky decision with a long delay, which is different from the existing studies on inter-temporal decision with risk. The main difference is that both the delay and probability of starting a business are uncertain, whereas the delay of other investments such as variable rate debt instrument is generally certain. However, the literature on inter-temporal risky choice related to the real situation is almost still in a blank state. In this study, we will conduct experiments related to both risky choice and inter-temporal choice in a specific situation – to start a business or get a job after graduation.

#### Future Self-Continuity and Inter-Temporal Risky Choice

The study of self-identity has developed into a topic that scholars have been paying attention to for a long time. Individuals’ self-identity could be extended from the past to the future, in which the self can be distinguished into the past self, the present self, and the future self (Welch-Ross, 2001). Among many studies, Hershfield et al. (2009) paid the most attention to the connection between the present self and the future self. The concept of FSC is proposed by Hershfield et al. (2009) to express individuals’ understanding of the continuity and consistency of their present and future selves. As the research progressed, Hershfield et al. (2011) summarized the FSC into three characteristics: similarity, which represents the similarity between the future self and the present self; vividness, the individual’s imagination of their future selves; and positivity, the degree to which people have a positive attitude toward their future selves. The higher the level of these three characteristics, the stronger the FSC (Hershfield et al., 2011).

Researchers have pushed up the FSC in different fields. This includes decision-making (e.g., Hershfield et al., 2009; Bartels and Rips, 2010; Bartels and Urminsky, 2011), consumption (Zhang and Aggarwal, 2015), and stress and mood (Rozental and Carlbring, 2014). In terms of choice, the research of FSC mainly focuses on inter-temporal choice. Hershfield et al. (2009) initially found that individuals with higher FSC had a larger number of later choices on the temporal discounting task and greater lifetime accumulation of financial assets in a community (after controlling for age and education). Subsequently, many studies have found that FSC can predict the time discount of inter-temporal choice in terms of savings and rewards. Individuals with higher FSC show lower time discount, that is, they will choose to wait for greater returns (Bartels and Rips, 2010). Meanwhile, through writing tasks (Hershfield et al., 2009), reading tasks (Bartels and Rips, 2010), and interactive tasks (Hershfield et al., 2011; Blouin-Hudon and Pychyl, 2016), the FSC of subjects can be changed. By increasing the degree of the psychological connection between the individual’s future self and the present self, it can be guided to make a choice that is beneficial to the future (Bartels and Urminsky, 2011).

To sum up, previous studies related to FSC and inter-temporal choice mainly focus on deterministic returns such as savings and rewards, while few studies focus on risks such as entrepreneurship. What is less clear is how people’s FSC and tenacity come together to influence inter-temporal risky choices. In this study, the inter-temporal choice and the risky choice are combined in the entrepreneurial context to explore the role of tenacity and FSC on the inter-temporal risky choice, so as to enrich the knowledge on personality, choice, and entrepreneurship. There are three aspects:

(1) The purpose of this article is to study the impact of tenacity on entrepreneurial choice, including the effect of tenacity on both risky choice and inter-temporal risky choice. Tenacity and risk always interact in entrepreneurial situations (Tadajewski and Jones, 2017). Visionary entrepreneurs are always willing to take a certain risk and stick with it in uncertainly circumstances. Their tenacity is reflected in their willingness to challenge themselves, take greater risks, and seek higher expected returns than their peers who chose to be employees. Therefore, it can be inferred that individuals with high tenacity are less likely to turn down a risk-return opportunity and are more likely to make entrepreneurial choices as a result of given conditions. The hypotheses are as follows: The higher degree of tenacity, the greater the likelihood of risky choice in the entrepreneurial context (H1); the higher the degree of tenacity, the greater the likelihood of inter-temporal risky choice in the entrepreneurial context (H2).
(2) Considering the entrepreneurial context where inter-temporal choice includes risk factors, the relationship between FSC and inter-temporal risky choice will be certified. Starting a business is a long-term commitment that the return often has to wait several years. The decision of starting a business is partly reflected in the inter-temporal choice. Based on the research of FSC, individuals who have a continuous and consistent cognition of themselves in each stage should make their choice beneficial to the future. People’s preferences in different decision-making fields are not completely consistent (Hardisty and Weber, 2009; Weatherly et al., 2010). Some literatures believe that risk decision-making is similar to inter-temporal decision-making (e.g., Rachlin et al., 1991; Keren and Roelofsma, 1995; Baucells and Heukamp, 2010), because similar functional forms are assumed for risk discounting (i.e., the value of an income will decrease with the probability decrease) and time discounting (i.e., the value of an income will decrease with the time delay) (Vanderveldt et al., 2015). Therefore, FSC should be positively correlated with inter-temporal risky choices. In other words, individuals whose perceptions of their future selves are more closely related to the present self are likely to wait longer for a greater return on risk. The hypothesis is as follows: The higher the degree of FSC, the greater the likelihood of inter-temporal risky choice in the entrepreneurial context (H3).
(3) To examine the reciprocal effect of FSC and tenacity on inter-temporal risky choice, we make the competing hypotheses as follows: FSC will mitigate the impact of tenacity on inter-temporal risky choice in the entrepreneurial context (H4a); FSC will amplify the impact of tenacity on inter-temporal risky choice in the entrepreneurial context (H4b).

## Materials and Methods

### Participants

Undergraduates from Beijing University of Posts and Telecommunications participated in this procedure. In the initial sample of 144 responses, 3 subjects are left out of the initial sample because of the inconsistency of their answers of the lie-detector items and 12 subjects are excluded from the analysis because of manipulation check. The valid participants have a total of 129 undergraduates. Among the remaining 129 students, 60 (46.51%) were males and 69 (53.49%) were females. The average age of students was 20.23 years (SD = 0.750, range from 18 to 22). In the sample, students were from various important subject areas, including business (51.16%), sociology (15.51%), sciences (2.32%), and engineering (31.01%). Each subject received 2 RMB as payment.

### Procedure

The questionnaire of this study consists of six parts: demographic survey, emotion scale, tenacity scale, FSC scale, and entrepreneurial choice with inter-temporal return as well as inter-temporal risk-return. An emotional scale is required to find out whether a subject’s mood is stable at the time of the survey. The study received the consent of the undergraduate students. After the demographic survey, subjects successively completed the psychometry of tenacity, risky choice task, future-self continuity measure, and at last the risky inter-temporal choice task.

It should be noted that there are only simple measurements on tenacity in recent quantitative studies such as a five-point scale with a few questions for a single factor. What constitutes tenacity and how to assess the diversity of people’s tenacity remain almost a blank on entrepreneurship. We drew on the health psychology study of hardiness. The term “hardiness” is used in the field of health to describe an individual’s tenacity in fighting disease. “Hardiness” refers to the state that an individual remains healthy in the face of stressful events, which is related to positive attitudes and coping styles (Kobasa, 1979). When the term “Hardiness” was first proposed by Kobasa (1979), it contained three elements: control, commitment, and challenge. The common characteristics of these three factors are largely consistent with the tenacity personality described and identified by most researchers, which have been extended to many other areas, such as the hardiness of college students under study pressure. Compared with hardiness, entrepreneurs’ tenacity contains the pursuit of self-value, the endurance of setbacks, and environmental uncertainty. For this, the differences in behavior patterns under different cultural contexts are taken into account; the hardiness scale of Chinese college students proposed by Tang (2008) is used to measure the tenacity of subjects in this survey. The scale used in this study divides tenacity into five factors: endurance, commitment, challenge, self-control, and control. This scale has been widely accepted by Chinese researchers.

The questionnaire was distributed and collected through the survey website Sojump and can be found in Chinese at the following link: https://www.wjx.cn/hm/cpcwc4b07k6voejxvveyqa.aspx. Sojump is the largest online survey platform in China. Since its launch in 2006, over 85.87 million questionnaires and 6.79 billion responses have been collected on Sojump.

### Instruments

#### Tenacity

The *Hardiness Scale* designed by Tang (2008) is used to assess the tenacity in the present study. This questionnaire is based on the characteristics of Chinese culture and widely quoted in China. The questionnaire measures the degree of tenacity on five dimensions (i.e., Endurance, Commitment, Challenge, Self-Control, Control) with 25 trails and 3 lie-detector items. Each dimension has four to seven items. An example of items is “In the face of difficulties, I usually spare no effort to overcome the difficulty.” Subjects were asked for each of the five dimensions for an answer that was described on a five-point Likert scale ranging from 1 (strongly disagree) to 5 (strongly agree). The scores for each dimension (i.e., Endurance, Commitment, Challenge, Self-Control, Control) can be summed up by the aggregation item scores, and the score of tenacity is the sum of these five dimensions. A high score implies a high degree of tenacity. In this study, the total internal coefficient alpha is 0.784, and the internal consistency reliability of five dimensions is 0.708 (the commitment subscale), 0.741 (the self-control subscale), 0.699 (the control subscale), 0.672 (the endurance capacity subscale), and 0.585 (the challenge subscale), respectively.

#### Future Self-Continuity

The *Future Self-Continuity Measure* adopts the scale created by Hershfield et al. (2009), with two featured questions. Each question has a graph with seven pairs of circles, in which the two circles range from completely not overlapping to completely overlapping, describing the degree of correlation between the current self and the self in the next decade. The scale made by Hershfield et al. (2009) is widely used for its high retest reliability. In Hershfield et al. (2009), the retest reliability presents at a high level: similarity (*r* = 0.66, *p* < 0.001; α = 0.79) and connectedness (*r* = 0.66, *p* < 0.001; α = 0.80). In order to help the subjects understand the concept of FSC, after answering the first question about future self-similarity, they are asked to imagine and write out their future selves in the next 10 years, then the scale is used to measure the degree of correlation between the present self and the future self. The higher the score, the higher the degree of FSC. This means the individuals tend to perceive the current self and the future self as a whole. This scale also shows an acceptable psychometrics property on reliability in this study. The individuals with higher FSC index had more vivid and positive images of future selves, while individuals with lower FSC index had less imaginative and negative subjective descriptions of their future selves.

#### Risky Choice Task

To simulate risky choice in an entrepreneurship scenario, participants read the following instructions before answering the questions:

“In this study, you were assumed to be a college graduate facing the decision between starting your own business and going into employment. Please choose from each of the following scenarios. All of the assumed income in the options are after-tax income.”

Subsequently, participants are presented with five questions and make a choice between business and job, with no correlation between the data of each option (see Supplementary Appendix 1). The salary for a job ranges from 240,000 to 800,000 RMB, and the income for a business ranges from 120,000 to 2 million RMB. The five options are ranked in the probability of return on risk. Option I has the highest risk-return probability. Option V has the lowest probability of risk-return. The probability that the business income is higher (lesser) than the salary for a steady job varies from 10 (0) to 90% (60%). Both the income and the salary are described as the earnings in the next 3 years under the hypothetical scenario. In other words, the title is described as the following: Employment, the expected salary in the next 3 years is “X”; Setting up a business, there is a “a%” chance of making a net profit of “*R*_1_” and a “b%” chance of making a net profit of “*R*_2_” in the next 3 years. For example, “Employment, the expected salary in 3 years is 240,000 RMB; Setting up a business, there is a 90% chance of making a net profit of 300,000 RMB, and a 10% chance of making a net profit of 120,000 RMB in 3 years.”

#### Risky Inter-Temporal Choice Task

*The Risky Inter-Temporal Choice Task* was compiled based on the entrepreneurial scenario. In order to promote the recognition of the opportunity and risk contained in the start-up, participants are asked to read the following instructions before answering the questions:

“You’re graduating from college soon, and the prospect of your career is promising to find a satisfying job even if you do not start a business. However, your university is encouraging college students to start their own businesses by providing some financial support and places. What’s more, you have learned the course on entrepreneurship. Based on the above, start a business means higher returns in the future, but you will face more challenges and uncertainties. On the other hand, getting a job implies a steady salary, but it may be less than the business. In that case, which one would you like to choose?”

After that, participants completed eight risky inter-temporal choices (see Supplementary Appendix 2). Each pair of options includes a certain annual salary and an uncertain delayed return. Among the options, the salary is fixed, which is described as the following: “A job with an annual salary of 110,000 RMB in the first year (the salary is paid at the end of the year). The salary increases by 10% every year for the next 4 years. The 5-year cumulative return is RMB 617,600. The total discounted return (the present value on the day you started working) is RMB 500,000.” For a business, the first 4 years are set as risk-free return, and the fifth year is set as risk-return. The eight options are ranked according to the degree of delay and uncertainty. There are three scenarios for the initial earning year: the third year, the fourth year, and the fifth year. The probability of return in year 5 is (1) *R*_1_ with a 70% chance and *R*_2_ with a 30% chance (*R*_1_ > *R*_2_) and (2) a 50% chance of *R*_1_ and a 50% chance of *R*_2_ (*R*_1_ > *R*_2_). Option I is as follows: A predictable venture, which is not profitable for the first 2 years, will make a profit of 100,000 yuan by the end of the third year, 200,000 yuan by the end of the fourth year, and a 70% (30%) chance of making a profit of 500,000 (400,000) yuan by the end of the fifth year.

### Manipulation Check

During the data processing, in order to eliminate the incorrect results caused by subject inattention, we checked the choice against common sense. The data is excluded in the following two cases. First, inconsistent answers are presented in the selection process of different probability. For example, in the condition of starting a business, a participant prefers a 50% chance of getting 800,000 to a 70 percent chance of getting 800,000. Second, inconsistent answers are given in the selection process of different amount. For example, if a participant prefers 100,000 rather than 200,000, the response violated dominance in choices. Therefore, 12 subjects were excluded from this process.

## Results

### Preliminary Analyses

The results of descriptive analysis are shown in Table 1. The effective number of participants in Task 1 is 129: all of them enter the statistical analysis. In Task 2, in addition to those who failed to pass the manipulation check, both 31 subjects who chose employment among all options and 5 subjects who chose start-up are deleted, following previous studies of Hershfield et al. (2009) and Kirby and Marakovic (1996). After deletion, the remaining 85 subjects meet the requirements. The Shapiro–Wilk test shows that the variables related to the tenacity and the FSC all follow the law of normal distribution (*p* > 0.05). The mean of Tenacity is 9.341 (SD = 10.71; Min = −19; Max = 35). Components of Tenacity can also be seen in Table 1. The mean of the FSC is 4.729, and the median is 5, indicating that most of the subjects believed that there is a relative consistency between the current self and the future self.

**TABLE 1:** Means, medians, standard deviations (SD), minimums (Min), and maximums (Max) of the study’s variables.

**Variables**	**Mean**	**Median**	**SD**	**Min**	**Max**
Tenacity	9.341	8.000	10.71	−13.00	35.00
Endurance	10.79	11.00	2.896	1.000	17.00
Commitment	20.57	21.00	3.583	9.000	28.00
Challenge	2.008	2.000	2.210	−3.000	7.000
Control	−17.88	−18.00	4.092	−30.00	−6.000
Self-control	−6.147	−6.000	2.982	−14.00	1.000
FSC	4.729	5.000	1.238	1.000	7.000
RRC	1.195	1.279	0.150	1.000	1.416
IRRC	1.249	1.280	0.095	1.000	1.341

**N* = 129. FSC, future Self-continuity; RRC, risk-return Coefficient; IRRC, inter-temporal risk-return coefficient.*

Table 2 reports the values of RRCs (risk-return coefficients) in Task 1 and IRRCs (inter-temporal risk-return coefficient) in Task 2, respectively. The indicator RRC is equal to the ratio of the expected return of entrepreneurial to the salary of employment. The indicator IRRC represents the risk-return coefficient after time discounting, derived to the ratio of the present value of risk-return to the certain income. The discount rate of employment and start-up is uniformly 10%, so as the present value of salary is 500,000 RMB. For entrepreneurial choice, the expected return of the fifth year is equal to the probability-weighted income. Both risk discount factor (RDF) and inter-temporal risk discount factor (IRDF) are also presented in Table 2. The RDF is equal to 1 over RRC, as well as the IRDF, which are presented in the research of Sun and Li (2010). However, given the complexity of the challenges and duration of uncertainties in start-up, the return on risk is more appropriate to be used to evaluate a business, compared to the risk-discounting factor. The risk-return coefficient is devoted to the subsequent analysis of this study.

**TABLE 2:** Risk-return coefficients and risk-discount factors of all options.

**Risky choice options**	**RRC**	**RDF**
I	1.175	0.851
II	1.467	0.682
III	1.407	0.711
IV	1.100	0.909
V	1.375	0.727

**Inter-temporal risky choice options**	**IRRC**	**IRDF**

I	0.893	1.120
II	0.916	1.092
III	1.027	0.973
IV	1.118	0.895
V	1.490	0.671
VI	1.671	0.599
VII	1.693	0.591
VIII	1.919	0.521

**N* = 129. RRC, risk-return coefficient; IRRC, Inter-temporal risk-return coefficient; RDF, risk-discount factor; IRDF, inter-temporal risk-discount factor.*

### Correlations Analysis

The pairwise correlation analysis of variables of tenacity and risky choices is reported in Table 3. Tenacity and RRC are positively correlated at a significance level of 5% (β = 0.179, *p* = 0.042), which partially confirms H1. It also formulates that Tenacity correlates with the risky choices in Task 1. It is significantly correlated with Options IV (β = 0.257, *p* = 0.003) and V (β = 0.216, *p* = 0.014) but not related to risky options I, II, III, and V. Specifically, three factors of tenacity (i.e., Endurance, Commitment, Challenge), predicting the participants’ risky choice behavior, are significantly correlated with RRC and risky options IV and V.

**TABLE 3:** Correlations among the RRC, risky choices the tenacity variables.

**Variables**	**RRC**	**Risky choice**				
		**I**	**II**	**III**	**IV**	**V**
Tenacity	0.179*	0.023	0.034	0.110	0.257**	0.216*
	(0.042)	(0.798)	(0.706)	(0.215)	(0.003)	(0.014)
Endurance	0.229**	0.169^#^	0.117	0.233**	0.289***	0.231**
	(0.009)	(0.056)	(0.187)	(0.008)	(0.000)	(0.009)
Commitment	0.236**	0.098	0.108	0.165^#^	0.283**	0.190*
	(0.007)	(0.268)	(0.222)	(0.062)	(0.001)	(0.031)
Challenge	0.152	0.070	0.045	0.082	0.222*	0.218*
	(0.086)	(0.432)	(0.613)	(0.357)	(0.011)	(0.013)
Control	0.035	−0.105	−0.009	0.028	0.114	0.101
	(0.695)	(0.237)	(0.917)	(0.755)	(0.198)	(0.255)
Self-control	−0.024	−0.108	−0.144	−0.128	−0.019	0.023
	(0.789)	(0.223)	(0.104)	(0.147)	(0.828)	(0.795)

*N* = 129. RRC, risk-return coefficient. *p < 0.05, **p < 0.01, ***p < 0.001 (two-tailed).

Table 4 reports the result from regression analysis of Model 1. The coefficient on Tenacity is positive and significant (*t* = 2.12) for the regression, suggesting that the tenacity of subjects affects their risky choices. By examining the core factors of Tenacity (i.e., Endurance, Commitment, Challenge), the explanatory power of the model in Table 5 has improved (*R*-squared increases from 0.032 to 0.064), the coefficient of Tenacity_DF (only includes its dominant factors in this study: Endurance, Commitment, Challenge) has become 0.005, and the significance level is 1% (*t* = 3.02).

**TABLE 4:** Regression analysis of tenacity to RRC.

**Variables**	**Coefficient**	**SE**	***T*-value**
Tenacity	0.003**	0.001	2.12
*R*^2^	0.032		

*N* = 129. ** represents that it is significant at 5%.

**TABLE 5:** Regression analysis of the core elements of tenacity to RRC.

**Variables**	**Coefficient**	**SE**	***T*-value**
Tenacity_DF	0.005***	0.002	3.02
*R*-squared	0.064		

*N* = 129. Tenacity_DF = Endurance + Commitment + Challenge. *** represents that it is significant at 1%.

### The Reciprocal Effect

**FIGURE 1:**
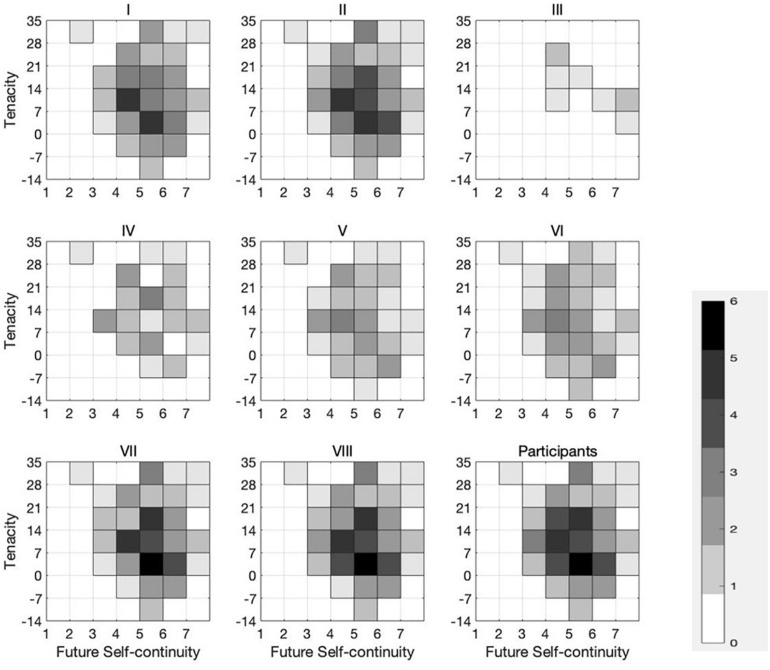
Relationship between tenacity, future self-continuity, and inter-temporal risky choice (*N* = 85).

To examine the FSC affecting a certain income (coded 0) or an uncertain return (coded 1) in the inter-temporal decision trials, the *T*-test is applied in Table 6. The intergroup differences of FSC are statistically significant for Options I (Dif. = 0.471, *p* = 0.081), II (Dif. = 0.827, *p* = 0.081), VII (Dif. = 1.360, *p* = 0.023), and VIII (Dif. = 0.943, *p* = 0.019). This partially suggests that FSC affects larger-later risky choices. It is quite clear that there is a requirement to analyze not only the effect of FSC on the inter-temporal choice in the decision to start a business but also the impact of tenacity on the risky choice and the interaction between the two factors. For this reason, an intuitive analysis is listed in Figure 1 before a more complex model is proposed.

**TABLE 6:** Difference significance of the FSCs in the inter-temporal risky choices.

**Inter-temporal risky choice**	**FSC of subjects**		**FSC of subjects who**		**Difference**	
	**choose start-up**		**who choose job**		**of FSC**	
	**Num.**	**Mean**	**Num.**	**Mean**	**Dif.**	***p*-value**
I	68	4.824	17	4.353	0.471	0.081
II	75	4.827	10	4.000	0.827*	0.023
III	9	4.778	76	4.723	0.054	0.451
IV	33	4.909	52	4.615	0.294	0.145
V	46	4.761	39	4.692	0.069	0.401
VI	51	4.765	34	4.676	0.089	0.375
VII	74	4.905	11	3.545	1.360***	0.000
VIII	77	4.818	8	3.875	0.943*	0.019

*N* = 85. *p < 0.05, ***p < 0.001.

Figure 1 shows the distribution statistics of entrepreneurial choices of 85 subjects under the two factors: self-continuity and tenacity. The darker the color, the more numbers for the entrepreneurial decision. In Options I, II, VII, and VIII, the entrepreneurial choices are saliently concentrated to the upper right. Furthermore, those with a high score of tenacity and self-continuity made fewer entrepreneurial choices in Options III, IV, V, and VI than those in Options I, II, VII, and VIII, but they are still more likely than other subjects to make entrepreneurial choices.

To explore the reciprocal effect of FSC and tenacity on entrepreneurial decision-making, we perform a least-square regression analysis and report the results in Table 7. This is done using Stata 11. At first, the explanatory power of Model 2 is significantly improved (adjusted *R*^2^ = 10.1%), compared to Model 1 (adjusted *R*^2^ = 3.2%). Then, the coefficient on Tenacity (β = 0.035, *t* = 2.88, *p* < 0.001) is significantly positive as expected in H2, indicating that subjects with hardiness personality in general made more larger-later choices and are willing to take a risk for a start-up. There is also a significant positive effect of *FST* (β = 2.130, *t* = 8.94, *p* < 0.001) which supports H3. The possibility of making entrepreneurial decisions increases with the increase in the degree of correlation between the current self and the future self. Consistent with our H4a, the coefficient on *FST* × *Tenacity* is significantly negative in the regression (β = −0.002, *t* = −3.12, *p* < 0.001).

**TABLE 7:** Regression analysis of tenacity and FSC to IRRC.

**Variables**	**Coefficient**	**SE**	***T*-value**
FSC	0.035***	0.112	2.88
Tenacity	0.009***	0.003	2.99
Tenacity × FSC	−0.002***	0.001	–3.12
*R*^2^	0.101		

*N* = 85. *** represents that it is significant at 1%.

Specifically, when we replaced *Tenacity* with *Tenacity_DF* (only includes its dominant factors in this study: Endurance, Commitment, Challenge), the explanatory power of the model is further improved (adjusted *R*^2^ = 11.5%), and the coefficients are larger in Table 8.

**TABLE 8:** Regression analysis of Tenacity_DF and FSC to IRRC.

**Variables**	**Coefficient**	**SE**	***T*-value**
Tenacity_DF	0.017***	0.005	3.81
FSC	0.134***	0.034	3.93
Tenacity_DF × FSC	−0.003***	0.001	–3.97
*R*-squared	0.115		

*N* = 85. Tenacity_DF = Endurance + Commitment + Challenge. *** represents that it is significant at 1%.

## Discussion

### Implications of Results

#### Tenacity and Risky Choice

In Model 1 and Model 2, the tenacity shows us a prominent positive effect on risky choices and inter-temporal risky choices. The results support H1 and H2. Individuals with high tenacity are more likely to take the venture, which is in fact an inter-temporal risk decision. It needs to span several years, which is different from the classical inter-temporal choice model that only spans several months. Meanwhile, the current study is not to reveal the relationship between tenacity and risk in start-up growth but to discover the trait that drives college students to make this choice. In this regard, individuals with tenacity are likely to endure the pressures and setbacks of the process and ultimately succeed in reaping the benefits. Therefore, they have the willingness and confidence to make entrepreneurial choices. The explanations of results in this present study on the risky choice-related entrepreneurial activity are consistent with several existing studies. Baum and Locke (2004) made a primary study in this field and found the positive effects of tenacity on the start-up process. Tadajewski and Jones (2017) argued that to be a pioneer in marketing, considerable tenacity for risk is needed. Scotter and Garg (2019) examined the trait of a new venture creation, suggesting that tenacity impacts entrepreneurial persistence behavior in different industry contexts. As an extension and enrichment of the existing conclusions, we provide knowledge to understand the relationship between tenacity and risks under a venture context, based on the perspective of the entrepreneurial choices of college students.

#### Tenacity and Inter-Temporal Risky Choice

The article also explores the effects of the five factors of tenacity on inter-temporal risk decision-making. The results showed that endurance, commitment, and challenge played a dominant role in influencing the choice of inter-temporal risk. They are shown in Tables 3, 5, and 8. In the pairwise correlation analysis, it can be seen that endurance, commitment, and challenge are related to several risky choices (Options III, IV, V). In regression analysis, the explanatory power of Model 1 and the significance of tenacity increased when it included only these three dominant factors. The explanatory power of Model 2 with Tenacity as the explanatory variable is mentioned as 11.5%, compared to 10.1% in Table 7. All of these showed that commitment, endurance, and challenge are robustly positive predictors of the risky choices. This implies that commitment, endurance, and challenge are the main factors that support individuals to build tenacity and take an inter-temporal risky choice.

Firstly, commitment represents a state of focused energy devoted to its own cause. This state enables one to be free from external temptation and interference. This quality of commitment can prompt entrepreneurs to be more dedicated to their own business. Therefore, individuals with commitment characteristics are more likely to be tenacious and make entrepreneurial choices.

Secondly, endurance is positively correlated with the risk-return coefficient at a high level. This suggests that higher endurance, which contributes to increased tenacity, can strongly predict the positive effect of tenacity on entrepreneurial choices. In entrepreneurship, endurance is the tolerance to the complexity of the entrepreneurial situation, the patience to dig through the details of business such as management nightmare, and the ability to manage emotions – to suppress negative emotions and cultivate positive emotions in the face of conflict and stress. It shows that individuals who are longsuffering are more likely to take on the challenge of starting a business.

Thirdly, as a concrete manifestation of personality quality, challenge is intuitive in supporting peoples’ decision to choose start-up. The high challenge means that in the face of various pressures and difficulties in the entrepreneurial context, one can overcome the pressure and difficulties through the adaptation mechanism. By taking challenges as positive incentives, people’s tenacity can be consolidated, so as to improve the level of risk-taking.

These factors jointly support the formation of entrepreneurial tenacity, through accepting challenges, enduring hardships, meeting commitments, and other aspects. They set up the impact of tenacity on individuals’ risky choices, revealing the mechanisms of tenacity for a business. This study implements the future research proposed by Rauch and Frese (2007), which points out that tenacity is a crucial trait in entrepreneurship and its research value has not been put into fully realized. In the future, the internal structure of tenacity and the relationship between tenacity factors and external start-up pressures can be explored.

#### Tenacity, FSC, and Inter-Temporal Risky Choice

In terms of self-continuity, this paper examines the realistic situation of start-up where time delay and uncertainty coexist. Since Hershfield et al. (2009) found that FSC has an impact on inter-temporal decision-making, relevant literature has mainly focused on determining the trade-off between immediate return and delayed return. Considering the uncertainty of the long interval caused by the particularity of the entrepreneurial situation, this study combined inter-temporal factors with risky choice and examined the inter-temporal risky choice in regression analysis, showing the relationship between FSC and inter-temporal risky choices. As the results manifest, individuals with high FSC give greater weight to future risky return. Therefore, the guidance and improvement of FSC can assist individuals in grasping the opportunities for business and making insightful choices.

Congregating the functions of tenacity and FSC, we explored a relationship between tenacity, FSC, and inter-temporal risky choice. The results of multivariable regression analysis show us that individuals with a high degree of FSC may slightly mitigate the pursuit of risky return, which is associated with a high level of tenacity. As can be seen in Figure 1, most of the subjects who had both high tenacity and high FSC chose Option I or II (the present value of Option I is 446,500 yuan; 457,790 yuan for Option II), compared to Options III and IV. They seem more likely to seek a balance between the high excess risk-return and the low robust risk-return. This result leads us to further reflection. Combined with previous research, we found that this may have something to do with the fact that the psychological processing of two kinds of decision-making is not consistent in different situations. For example, in the context of inflation expectations, personality traits, and being a gambler or not (Myerson et al., 2003), there are differences in the effect of mental processing on inter-temporal and risky choice. So, it is also possible that in the entrepreneurial context, FSC plays a different role in risky choices and in inter-temporal choices. The effect is that when both are at a higher level, it does not lead to a pursuit for extremely higher inter-temporal risk-return but is more likely to contribute to the choice of medium-high risk-return. Sun and Li (2011) on inter-temporal choice with risks in the Chinese culture can also confirm this point of view.

Individuals with a high degree of FSC make choices in ways that they believe are better for their future selves. Hence, the one they choose may not necessarily be the highest risk-return in the inter-term risk scenario of start-up. It is their idea of what is best for the survival of the business. This is also consistent with the conclusion of Hershfield et al. (2009) that those with a higher FSC are more likely to make choices beneficial to the potential self. In their study, they found that individuals with a high FSC had more assets. Moreover, resident-owned assets are more likely to be acquired through sound investments such as savings and bonds than through riskier investments. These suggest that the nature of the high FSC involves a tendency to make the future favorable to one’s self at each stage in the long run.

On this basis, we suspect that FSC helps people develop a conservative mindset, paired with tenacity, for the purpose of achieving relatively long-term and sustainable return. Under the entrepreneurial situation, the performance is to seek smooth income. The pursuit of high risk-return guided by tenacity competes with the long-term return related to the future self in entrepreneurial activities. It, in turn, is more likely to result in relatively modest co-effects than outright high-risk activities.

### Limitations and Future Directions

There are some possible limitations of the present study that need to be acknowledged. The Tenacity Scale from Tang (2008) used in this study was developed for Chinese college students, so this discovery is based on data from Chinese college students. More evidence and testing are needed as to whether it applies to other age-group educational levels, as well as other contexts and other countries. However, it is likely that this relationship between tenacity and risk choice will also probably exist in other countries and regions.

We also know that the study design contains several risk factors (i.e., probability, time, amount) and seems a little complicated. However, the design of this study integrates previous studies on inter-temporal choice and the risky choice. Compared to previous studies, this study has two differences: 1) tasks are based primarily on risk choice, and 2) the time delay is longer and spans several years. This design is suitable for the complex entrepreneurial situation. It is set up to get relatively reliable results about the willingness for start-up. The complexity of the model may make it difficult to explain the interaction of factors (i.e., probability, time, amount) and the load of each factor. Therefore, we suggest carrying out a detailed division in the future and further study on inter-temporal risk choice.

The measurement of tenacity also needs discussing. The tenacity scale of this paper is a Hardiness Scale based on Chinese cultural background. The psychological term, “hardiness,” was first introduced by Kobasa (1979), initially including commitment, control, and challenge, which are mainly used in the research on physical and mental health (e.g., hypertension, heart disease and diabetes) under stressful events. When it comes into China, the inner strength of overcoming a serious illness for a long time was defined into “Jian Ren Xing,” which means a trait related to perseverance, high endurance, and unremitting self-management. Therefore, the generally accepted Chinese Hardiness Scale added two factors: endurance and self-control. It is a new scale consisting of five factors: commitment, control, challenge, endurance, and self-control. Thereupon, the term “Jian Ren Xing” in Chinese has come closer to the nature of tenacity than the term “hardiness” in English. This implication is supported by the findings of this paper that endurance is the most significant factor in relation to risky choice (Table 3). At the same time, self-control and control are not significant for the risky choices of start-up and employment. This may be partly because the scale is not designed directly for the tenacity of coping with stress in entrepreneurial situations, so the validity of some questions may be insufficient. In future, it is necessary to optimize the measurement tool of tenacity and develop a common tenacity scale based on the pressure situation of entrepreneurial development.

The effects of FSC can be further studied in the entrepreneurial context. Hershfield et al. (2009) pointed out that individuals who rated their future selves as more relevant to their current selves may have more vivid and positive images in the next decade, but we did not provide further insight into this. Namely, our findings fail to explain how different characteristics of FSC play a role in the choice. Hershfield et al. (2011) summarized the characteristics of FSC as similarity, vividness, and positivity. Future research can further explore the mechanism of these three Hershfield factors of FSC on both inter-temporal choices and risky choices in the entrepreneurial context through various methods.

## Conclusion

Considering the risky choice with a long-time delay in the entrepreneurial context, and based on the role of personality traits, our results show that tenacity has a positive effect on both risky and inter-temporal risky choice. This means that individuals with high tenacity can accept more uncertainty and wait longer in the entrepreneurial context. At the same time, the results show that endurance, commitment, and challenge play a major role in the impact of tenacity on entrepreneurial choice. It draws out the dominant factors from the five factors of tenacity in inter-temporal risk decision-making and suggests that intensive training in these three specific qualities could embody the tenacity of entrepreneurs.

Furthermore, the reciprocal effect demonstrates that FSC slightly mitigates the pursuit of risky return by subjects who have the high degree of tenacity. The individuals with high FSC and tenacity chose both the option of the lowest risk-return with a short delay (3 years) and the option of the highest risk-return with a long delay (5 years). It means that in the entrepreneurial context, FSC plays a different role in risky choices and in inter-temporal choices. It is important that FSC may help people develop a conservative mindset, paired with tenacity, in which the worthwhile goal is to seek a smooth income rather than pursue an extreme high risk-return. This implies that the conservative self-concept and the pursuit of risk-return guided by tenacity compete and reinforce each other in the entrepreneurial activities. These findings extend the existing knowledge of the personality, choice, and entrepreneurship.

## Data Availability Statement

All datasets presented in this study are included in the article/Supplementary Material.

## Ethics Statement

Ethical approval was not provided for this study on human participants because an ethics approval was not required as per applicable institutional and national guidelines and regulations. The informed consent of the participants was implied through survey completion.

## Author Contributions

XZ and YO designed the questionnaire, carried out the experiments, and wrote the manuscript. XZ analyzed the experimental results. Both authors contributed to the article and approved the submitted version.

## Conflict of Interest

The authors declare that the research was conducted in the absence of any commercial or financial relationships that could be construed as a potential conflict of interest.

## References

[B1] Ahlbrecht M. Weber M. (1997). An empirical study on intertemporal decision making under risk. *Manag. Sci.* 43 813–826. 10.1287/mnsc.43.6.813 19642375

[B2] Anderson L. R. Stafford S. L. (2008). Individual decision-making experiments with risk and intertemporal choice. *J. Risk Uncertain.* 38 51–72. 10.1007/s11166-008-9059-4

[B3] Ariely D. (2009). *Predictably Irrational: The Hidden Forces That Shape Our Decisions.* New York, NY: Harper Perennial.

[B4] Bartels D. M. Rips L. J. (2010). Psychological connectedness and intertemporal choice. *J. Exp. Psychol. Gen.* 139 49–69. 10.1037/a0018062 20121312

[B5] Bartels D. M. Urminsky O. (2011). On intertemporal selfishness: how the perceived instability of identity underlies impatient consumption. *J. Consum. Res.* 38 182–198. 10.1086/658339

[B6] Bass B. M. Stogdill R. M. (1990). *Bass & Stogdill’s Handbook of Leadership: Theory, Research, and Managerial Implications.* New York, NY: Free Press.

[B7] Baucells M. Heukamp F. H. (2010). Common ratio using delay. *Theory Decis.* 68 149–158. 10.1007/s11238-008-9130-2

[B8] Baum J. R. Locke E. A. (2004). The relationship of entrepreneurial traits. skill, and motivation to subsequent venture growth. *Appl. Psychol.* 89 587–598. 10.1037/0021-9010.89.4.587 15327346

[B9] Blouin-Hudon E.-M. C. Pychyl T. A. (2016). A mental imagery intervention to increase future self-continuity and reduce procrastination. *Appl. Psychol.* 66 326–352. 10.1111/apps.12088

[B10] Brandstätter E. Gigerenzer G. Hertwig R. (2006). Risky choice with heuristics. *Psychol. Rev.* 115 281–289. 10.1037/0033-295X.115.1.281 18211205

[B11] Carvalho L. S. Meier S. Wang S. W. (2016). Poverty and economic decision-making: evidence from changes in financial resources at payday. *Am. Econ. Rev.* 106 260–284. 10.1257/aer.20140481 28003681 PMC5167530

[B12] Desti K. Kumar N. S. (2008). “Drivers of entrepreneurial performance of Indian entrepreneurs in Singapore,” in *Proceedings of the Book of Abstracts of the 2008 Singapore Management University Edge Conference*, Singapore, 24–27.

[B13] Ekpoh U. I. Edet A. O. (2011). Entrepreneurship education and career intentions of tertiary education students in Akwa Ibom and Cross River States, Nigeria. *Int. Educ. Stud.* 4 172–178. 10.5539/ies.v4n1p172

[B14] Epper T. Fehr-Duda H. Bruhin A. (2011). Viewing the future through a warped lens: why uncertainty generates hyperbolic discounting. *J. Risk Uncertain.* 43 169–203. 10.1007/s11166-011-9129-x

[B15] Green L. Myerson J. (2004). A discounting framework for choice with delayed and probabilistic rewards. *Psychol. Bull.* 130 769–792. 10.1037/0033-2909.130.5.769 15367080 PMC1382186

[B16] Hardisty D. J. Weber E. U. (2009). Discounting future green: money versus the environment. *J. Exp. Psychol. Gen.* 138 329–340. 10.1037/a0016433 19653793

[B17] Hershfield H. E. Garton M. T. Ballard K. Samanez-Larkin G. R. Knutson B. (2009). Don’t stop thinking about tomorrow: individual differences in future self-continuity account for saving. *Judge. Decis. Making* 4 280–286. 10.7312/barb14438-003 PMC2747683 19774230

[B18] Hershfield H. E. Goldstein D. G. Sharpe W. F. Fox J. Yeykelis L. Carstensen L. L. (2011). Increasing saving behavior through age-progressed renderings of the future self. *J. Mark. Res.* 48 S23–S37. 10.1509/jmkr.48.spl.s23 24634544 PMC3949005

[B19] Hou F. Su Y. Lu M. Qi M. (2019). Model of the entrepreneurial intention of university students in the Pearl River Delta of China. *Front. Psychol.* 10:916. 10.3389/fpsyg.2019.00916 31114522 PMC6503157

[B20] House R. J. Shamir B. (1993). “Toward the integration of transformational, charismatic and visionary theories of leadership,” in *Leadership Theory and Research: Perspectives and Directions*, eds Chemers M. Ayman R., (San Diego, CA: Academic Press), 81–107.

[B21] Jones B. A. Rachlin H. (2009). Delay, probability, and social discounting in a public goods game. *J. Exp. Anal. Beha.* 91 61–73. 10.1901/jeab.2009.91-61 19230512 PMC2614818

[B22] Keren G. Roelofsma P. (1995). Immediacy and certainty in intertemporal choice. *Organ. Behav. Hum. Dec. Process.* 63 287–297. 10.1006/obhd.1995.1080

[B23] Kirby K. N. Marakovic N. N. (1996). Delay-discounting probabilistic rewards: rates decrease as amounts increase. *Psychon. Bull. Rev.* 3, 100–104. 10.3758/BF03210748 24214810

[B24] Kobasa S. C. (1979). Stressful life events, personality, and health: an inquiry into hardiness. *J. Personal. Soc. Psychol.* 37 1–11. 10.1037/0022-3514.37.1.1 458548

[B25] Konstantinidis E. van Ravenzwaaij D. Guney S. Newell B. R. (2020). Now for sure or later with a risk? Modeling risky intertemporal choice as accumulated preference. *Decision* 7, 91–120. 10.1037/dec0000103

[B26] Krueger N. F. Brazeal D. V. (1994). Entrepreneurial potential and potential entrepreneurs. *Entrep. Theory Pract.* 18 91–104. 10.1177/104225879401800307

[B27] Lades L. K. (2012). Towards an incentive salience model of intertemporal choice. *J. Econ. Psychol.* 33 833–841. 10.1016/j.joep.2012.03.007

[B28] Laibson D. (1997). Golden eggs and hyperbolic discounting. *Q. J. Econ.* 112 443–478. 10.1162/003355397555253 32495221

[B29] Locke E. A. (2000). *The Prime Movers.* New York, NY: Amacom.

[B30] Loewenstein G. Prelec D. (1992). Anomalies in intertemporal choice: evidence and an interpretation. *Q. J. Econ.* 107 573–597. 10.2307/2118482

[B31] Mata R. Schooler L. J. Rieskamp J. (2008). The aging decision maker: cognitive aging and the adaptive selection of decision strategies. *Psychol. Aging* 22 796–810. 10.1037/0882-7974.22.4.796 18179298

[B32] Miller D. (2014). A downside to the entrepreneurial personality? *Entrep. Theory Pract.* 39 1–8. 10.1111/etap.12130

[B33] Murnieks C. Y. Cardon M. S. Sudek R. White T. D. Brooks W. T. (2016). Drawn to the fire: the role of passion, tenacity and inspirational leadership in angel investing. *J. Bus. Ventur.* 31, 468–484. 10.1016/j.jbusvent.2016.05.002

[B34] Myerson J. Green L. Scott H. J. Holt D. D. Estle S. J. (2003). Discounting delayed and probabilistic rewards: processes and traits. *J. Econ. Psychol.* 24 619–635. 10.1016/s0167-4870(03)00005-9

[B35] Öncüler A. (2000). *Intertemporal Choice Under Uncertainty: A Behavioral Perspective.* Working Paper 2000/37/TM, Fontainebleau: INSEAD.

[B36] Peterman N. E. Kennedy J. (2003). Enterprise education: influencing students’ perceptions of entrepreneurship. *Entrep. Theory Pract.* 28 129–144. 10.1046/j.1540-6520.2003.00035.x

[B37] Pihie Z. A. L. (2009). Entrepreneurship as a career choice: an analysis of entrepreneurial self-efficacy and intention of university students. *Eu. J. Soc. Sci.* 9 338–349.

[B38] Plambeck E. L. Wang Q. (2013). Implications of hyperbolic discounting for optimal pricing and scheduling of unpleasant services that generate future benefits. *Manage. Sci.* 59 1927–1946. 10.1287/mnsc.1120.1673 19642375

[B39] Prelec D. Loewenstein G. (1991). Decision making over time and under Uncertainty: a common approach. *Manage. Sci.* 37 770–786. 10.1287/mnsc.37.7.770 19642375

[B40] Rachlin H. C. Raineri A. Cross D. V. (1991). Subjective probability and delay. *J. Exp. Anal. Behav.* 55 233–244. 10.1901/jeab.1991.55-233 2037827 PMC1323057

[B41] Rauch A. Frese M. (2007). Let’s put the person back into entrepreneurship research: a meta-analysis on the relationship between business owners’ personality traits, business creation, and success. *Eu. J. Work Organ. Psychol.* 16 353–385. 10.1080/13594320701595438

[B42] Rozental A. Carlbring P. (2014). Understanding and treating procrastination: a review of a common self-regulatory failure. *Psychology* 5 1488–1502. 10.4236/psych.2014.513160

[B43] Samuelson P. A. (1937). A note on measurement of utility. *Rev. Econ. Stud.* 4 155–161. 10.2307/2967612

[B44] Scotter J. R. II Garg S. (2019). Entrepreneurial tenacity and self-efficacy effects on persisting across industry contexts. *Contemp. Manag. Res.* 15 147–173. 10.7903/cmr.19501

[B45] Shaver K. G. Scott L. R. (1992). Person, process, choice: the psychology of new venture creation. *Entrep. Theory Pract.* 16 23–46. 10.1177/104225879201600204

[B46] Sihombing S. O. (2012). Comparing entrepreneurship intention: a multigroup structural equation modeling approach. *Int. Res. J. Bus. Stud.* 5 57–71. 10.21632/irjbs.5.1.57-71

[B47] Sun S. Yao Z. Wei J. Yu R. (2015). Calm and smart? A selective review of meditation effects on decision making. *Front. Psychol.* 6:1059. 10.3389/fpsyg.2015.01059 26257700 PMC4513203

[B48] Sun Y. Li S. (2010). The effect of risk on intertemporal choice. *J. Risk Res.* 13 805–820. 10.1080/13669871003606224

[B49] Sun Y. Li S. (2011). Testing the effect of risk on intertemporal choice in the Chinese cultural context. *J. Soc. Psychol.* 151 517–522. 10.1080/00224545.2010.503719 21755658

[B50] Tadajewski M. Jones B. (2017). Autobiographical reflections part II: risk, tenacity and philosophies of research. *J. Hist. Res. Mark.* 9 210–216. 10.1108/jhrm-06-2017-0020

[B51] Tang Y. (2008). *A Primary Design of Hardiness Questionnaire of University Students.* Master Thesis, Northwest University, Xi’an.

[B52] Thaler R. H. (2008). Mental accounting and consumer choice. *Mark. Sci.* 27 15–25. 10.1287/mksc.1070.0330 19642375

[B53] Tian X. Wu Y. Wang Y. (2018). Career calling of nascent entrepreneurs in china: structure and measurement. *Soc. Behav. Pers. Int. J.* 46 695–704. 10.2224/sbp.6656

[B54] Vanderveldt A. Green L. Myerson J. (2015). Discounting of monetary rewards that are both delayed and probabilistic: delay and probability combine multiplicatively, not additively. *J. Exp. Psychol. Learn. Mem. Cogn.* 41 148–162. 10.1037/xlm0000029 24933696 PMC4268098

[B55] Wang S. M. Yueh H. P. Wen P. C. (2019). How the new type of entrepreneurship education complements the traditional one in developing entrepreneurial competencies and intention. *Front. Psychol.* 10:2048. 10.3389/fpsyg.2019.02048 31572260 PMC6753869

[B56] Weatherly J. N. Terrell H. K. Derenne A. (2010). Delay discounting of different commodities. *J. Gen. Psychol.* 137 273–286. 10.1080/00221309.2010.484449 20718227

[B57] Weber B. J. Chapman G. B. (2005). The combined effects of risk and time on choice: does uncertainty eliminate the immediacy effect? Does delay eliminate the certainty effect? *Organ. Beha. Hum. Dec. Process.* 96 104–118. 10.1016/j.obhdp.2005.01.001

[B58] Welch-Ross M. (2001). “Personalizing the temporally extended self: evaluative self-awareness and the development of autobiographical memory,” in *The Self in Time: Developmental Perspectives*, eds Moore C. Lemmon K., (Mahwah, NJ: Lawrence Erlbaum Associates), 97–120.

[B59] Wu W. Wang H. Lee H. Lin Y. Guo F. (2019). How machiavellianism, psychopathy, and narcissism affect sustainable entrepreneurial orientation: the moderating effect of psychological resilience. *Front. Psychol.* 10:779. 10.3389/fpsyg.2019.00779 31110485 PMC6499190

[B60] Zhang M. Aggarwal P. (2015). Looking ahead or looking back: current evaluations and the effect of psychological connectedness to a temporal self. *J. Consum. Psychol.* 25 512–518. 10.1016/j.jcps.2015.01.002

