# Supplementary material for: Entrepreneurship: Tenacity, Future Self-Continuity, and Inter-Temporal Risky Choice
Source: Front Psychol. 2020 Aug 7;11:1647. doi: 10.3389/fpsyg.2020.01647 (PMC7426468; doi:10.3389/fpsyg.2020.01647)
Supplement: Supplementary file 1 [file Data_Sheet_1.docx]

**Appendix 1.** **Risky Choice**

Participants completed the five risky choices during the risky task. The salary for a job ranges from 240,000 to 800,000 RMB, and the income for a business ranges from 120,000 to 2 million RMB. The five options are ranked in the probability of return on risk. Option I has the highest risk-return probability but the lowest risk-return. Option V has the lowest probability of risk-return but the highest risk-return. The probability that the business income is higher (lesser) than the salary for a steady job varies from 10 (0) to 90% (60%). Both the income and the salary are described as the earnings in the next 3 years under the hypothetical scenario.

|  | Expected salary in the next 3 years | Net profit of startup in the next 3years |
| --- | --- | --- |
| Option ① | ￥240，000 | 90% profit ￥300,000;  10% profit ￥120,000 |
| Option ② | ￥300，000 | 80% profit ￥500,000;  20% profit ￥200,000 |
| Option ③ | ￥270，000 | 60% profit ￥400,000;  40% profit ￥200,000 |
| Option ④ | ￥500，000 | 10% profit ￥1,000,000;  90% profit ￥500,000 |
| Option ⑤ | ￥800，000 | 40% profit ￥2,000,000;  60% profit ￥500,000 |
