# Supplementary material for: Entrepreneurship: Tenacity, Future Self-Continuity, and Inter-Temporal Risky Choice
Source: Front Psychol. 2020 Aug 7;11:1647. doi: 10.3389/fpsyg.2020.01647 (PMC7426468; doi:10.3389/fpsyg.2020.01647)
Supplement: Supplementary file 2 [file Data_Sheet_2.docx]

**Appendix 2. Risky Choice over Time**

Participants completed the eight risky choices over time during the risky inter-temporal choices task. Each pair of options includes a certain annual salary and an uncertain delayed start-up return. Among the options, the hypothetical salary is fixed, which is described as the following: “A stable job with an annual salary of 110,000 RMB in the first year (assuming the salary is paid at the end of the year). The salary increases by 10% every year for the next 4 years. The 5-year cumulative return is RMB 617,600. The total discounted return (the present value on the day you started working) is RMB 500,000.” For a business, the first 4 years are set as risk-free return, and the fifth year is set as risk-return. The eight options are ranked according to the degree of delay and uncertainty. There are three scenarios for the initial earning year: the third year, the fourth year, and the fifth year. The probability of return in year 5 is (1) R1 with a 70% chance and R2 with a 30% chance (R1 > R2) and (2) a 50% chance of R1 and a 50% chance of R2(R1 > R2).

| Option ① | | | | | |
| --- | --- | --- | --- | --- | --- |
|  | 1^st^ year | 2^nd^ year | 3^rd^ year | 4^th^ year | 5^th^ year |
| Employment | ￥110,000 | ￥121,000 | ￥132,100 | ￥146,400 | ￥161,100 |
| Startup | 0 | 0 | 100,000 | 200,000 | 70% profit ￥500,000  30% profit ￥400,000 |
| Option ② | | | | | |
|  | 1^st^ year | 2^nd^ year | 3^rd^ year | 4^th^ year | 5^th^ year |
| Employment | ￥110,000 | ￥121,000 | ￥132,100 | ￥146,400 | ￥161,100 |
| Startup | 0 | 0 | 100,000 | 200,000 | 50% profit ￥500,000  50% profit ￥400,000 |
| Option ③ | | | | | |
|  | 1^st^ year | 2^nd^ year | 3^rd^ year | 4^th^ year | 5^th^ year |
| Employment | ￥110,000 | ￥121,000 | ￥132,100 | ￥146,400 | ￥161,100 |
| Startup | 0 | 0 | 0 | 200,000 | 70% profit ￥1,500,000  30% profit ￥700,000 |
| Option ④ | | | | | |
|  | 1^st^ year | 2^nd^ year | 3^rd^ year | 4^th^ year | 5^th^ year |
| Employment | ￥110,000 | ￥121,000 | ￥132,100 | ￥146,400 | ￥161,100 |
| Startup |  |  |  | 200，000 | 50% profit ￥1,500,000  50% profit ￥700,000 |
| Option ⑤ | | | | | |
|  | 1^st^ year | 2^nd^ year | 3^rd^ year | 4^th^ year | 5^th^ year |
| Employment | ￥110,000 | ￥121,000 | ￥132,100 | ￥146,400 | ￥161,100 |
| Startup |  |  |  | 100，000 | 70% profit ￥1,000,000  30% profit ￥600,000 |
| Option ⑥ | | | | | |
|  | 1^st^ year | 2^nd^ year | 3^rd^ year | 4^th^ year | 5^th^ year |
| Employment | ￥110,000 | ￥121,000 | ￥132,100 | ￥146,400 | ￥161,100 |
| Startup |  |  |  | 100，000 | 50% profit ￥1,000,000  50% profit ￥600,000 |
| Option ⑦ | | | | | |
|  | 1^st^ year | 2^nd^ year | 3^rd^ year | 4^th^ year | 5^th^ year |
| Employment | ￥110,000 | ￥121,000 | ￥132,100 | ￥146,400 | ￥161,100 |
| Startup |  |  |  |  | 70% profit ￥3,000,000  30% profit ￥1,500,000 |
| Option ⑧ | | | | | |
|  | 1^st^ year | 2^nd^ year | 3^rd^ year | 4^th^ year | 5^th^ year |
| Employment | ￥110,000 | ￥121,000 | ￥132,100 | ￥146,400 | ￥161,100 |
| Startup |  |  |  |  | 50% profit ￥3,000,000  50% profit ￥1,500,000 |
